# Chronic Oral Infection with *Porphyromonas gingivalis* Accelerates Atheroma Formation by Shifting the Lipid Profile

**DOI:** 10.1371/journal.pone.0020240

**Published:** 2011-05-19

**Authors:** Tomoki Maekawa, Naoki Takahashi, Koichi Tabeta, Yukari Aoki, Hirotaka Miyashita, Sayuri Miyauchi, Haruna Miyazawa, Takako Nakajima, Kazuhisa Yamazaki

**Affiliations:** 1 Center for Transdisciplinary Research, Niigata University, Niigata, Japan; 2 Laboratory of Periodontology and Immunology, Division of Oral Science for Health Promotion, Niigata University Graduate School of Medical and Dental Sciences, Niigata, Japan; 3 General Dentistry and Clinical Education Unit, Niigata University Medical and Dental Hospital, Niigata, Japan; Fundació Institut Germans Trias i Pujol; Universitat Autònoma de Barcelona CibeRES, Spain

## Abstract

**Background:**

Recent studies have suggested that periodontal disease increases the risk of atherothrombotic disease. Atherosclerosis has been characterized as a chronic inflammatory response to cholesterol deposition in the arteries. Although several studies have suggested that certain periodontopathic bacteria accelerate atherogenesis in apolipoprotein E-deficient mice, the mechanistic link between cholesterol accumulation and periodontal infection-induced inflammation is largely unknown.

**Methodology/Principal Findings:**

We orally infected C57BL/6 and C57BL/6.KOR-*Apoe^shl^* (B6.Apoeshl) mice with *Porphyromonas gingivalis*, which is a representative periodontopathic bacterium, and evaluated atherogenesis, gene expression in the aorta and liver and systemic inflammatory and lipid profiles in the blood. Furthermore, the effect of lipopolysaccharide (LPS) from *P. gingivalis* on cholesterol transport and the related gene expression was examined in peritoneal macrophages. Alveolar bone resorption and elevation of systemic inflammatory responses were induced in both strains. Despite early changes in the expression of key genes involved in cholesterol turnover, such as liver X receptor and ATP-binding cassette A1, serum lipid profiles did not change with short-term infection. Long-term infection was associated with a reduction in serum high-density lipoprotein (HDL) cholesterol but not with the development of atherosclerotic lesions in wild-type mice. In B6.Apoeshl mice, long-term infection resulted in the elevation of very low-density lipoprotein (VLDL), LDL and total cholesterols in addition to the reduction of HDL cholesterol. This shift in the lipid profile was concomitant with a significant increase in atherosclerotic lesions. Stimulation with *P. gingivalis* LPS induced the change of cholesterol transport via targeting the expression of LDL receptor-related genes and resulted in the disturbance of regulatory mechanisms of the cholesterol level in macrophages.

**Conclusions/Significance:**

Periodontal infection itself does not cause atherosclerosis, but it accelerates it by inducing systemic inflammation and deteriorating lipid metabolism, particularly when underlying hyperlidemia or susceptibility to hyperlipidemia exists, and it may contribute to the development of coronary heart disease.

## Introduction

Coronary heart disease (CHD) is the leading cause of death in Japan and other developed countries. The major pathway underlying CHD pathology is atherosclerosis. Several risk factors for atherosclerosis have been identified, including smoking, hypertension, hyperglycemia, hypercholesterolemia and genetic factors. However, atherosclerosis can develop in the absence of these classic risk factors [1]. Recent epidemiological studies have suggested a link between atherosclerosis and infection/inflammation. Associations have been reported with *Chlamydia pneumoniae*, *Helicobacter pylori*, and cytomegalovirus [2], [3], as well as with dental infections, particularly those associated with periodontitis [4], [5].

Periodontitis is a chronic infectious disease initiated by a group of periodontopathic bacteria, such as *Porphyromonas gingivalis*. Periodontitis destroys the tooth-supporting tissues and leads to tooth loss if not adequately treated. Of the several hypotheses proposed to account for the effect of chronic infections on the development of atherosclerosis [6], one—the induction of low-grade systemic inflammation by periodontal infection—is particularly important. In fact, case-control and intervention studies [7], [8] have clearly demonstrated that high-sensitivity C-reactive protein (hs-CRP) and interleukin (IL)-6 protein levels are higher in cases of periodontitis and that successful treatment of periodontitis decreases the levels of both mediators. However, despite inferential evidence of an association between periodontitis and CHD, no precise underlying mechanism linking these two diseases has been elucidated.

Several animal studies have assessed the effects of periodontopathic bacterial infection on atherogenesis and documented the formation of atheromatous plaques and elevated systemic inflammatory markers [9], [10], [11]. Though, there are several points to consider before these findings can be extrapolated to link human periodontitis to atherosclerosis. First, intravenous inoculation with bacteria, rather than the natural route of human periodontitis (i.e., via oral tissues), was used in some experiments. Although transient bacteremia is common in association with the manipulation of the teeth and periodontal tissues, the magnitude of bacteremia resulting from a dental procedure is relatively low (<10^4^ colony-forming units (CFUs) of bacteria per milliliter) [12]. Moreover, there is no information about the relationship between the severity and extent of the disease and the magnitude of bacteremia. Therefore, the findings observed with direct inoculation of bacteria may not reflect the effects of naturally occurring periodontitis. Second, most studies used apolipoprotein E (ApoE)-deficient mice that spontaneously develop hyperlipidemia, which is a well-characterized risk factor for atherosclerosis. In patients with periodontitis, total cholesterol and low-density lipoprotein (LDL) cholesterol levels were increased and high-density lipoprotein (HDL) cholesterol levels were decreased compared to those in healthy subjects [13], [14], [15]. Therefore, it is reasonable to investigate the mechanism by which periodontal infection may affect and modify existing risk factors. However, the ways in which periodontal infection affects tissues and organs related to atherogenesis have not been elucidated, primarily because of the limitations of the experimental design described above and the use of short-term infection models rather than chronic infection models.

Here, we compared short-term and long-term infections in normocholesterolemic and hyperlipidemic mice. We found that oral inoculation of *P. gingivalis* affected the gene expression profiles in the aorta and liver, irrespective of abnormalities in the serum lipid profile. Additionally, infection accelerated the development of atheromatous plaques, and the serum lipid profile became more proatherogenic in the presence of preexisting hyperlipidemia.

## Results

### Alveolar bone loss due to oral Infection with *P. gingivalis*

**Figure 1:**
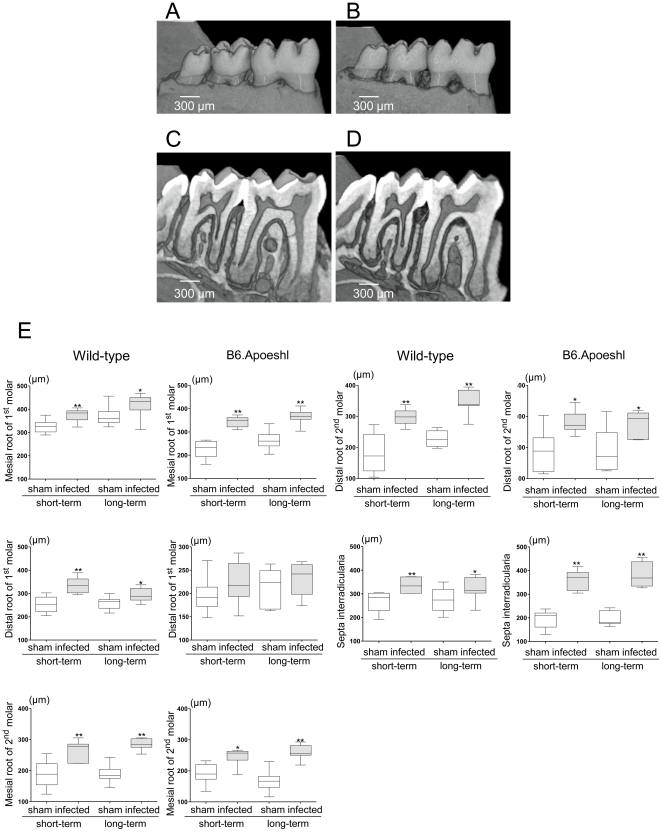
Determination of alveolar bone loss (A–D). Three-dimensional (A and B) and two-dimensional (C and D) microcomputed tomography images of representative samples from sham-infected mice (A and C) and infected mice (B and D) are presented. The infected group (A) showed an increase in the distance from the cement-enamel junction (CEJ) to the alveolar bone crest compared with the sham-infected group (B). Two-dimensional micro-CT sections for the infected group (C) but not the sham-infected group (D) showed alveolar bone loss at the bifurcation. Numerical analysis of alveolar bone loss between the control group and the infected group (E; N = 10 in each group) was performed. The distance between the cement-enamel junction and alveolar bone crest in each tooth root was determined from three-dimensional micro-CT images using image visualization software. Box plots present medians and the 25th and 75th percentiles as boxes and the 10th and 90th percentiles as whiskers. Significant differences were observed between the infected group and the sham-infected group (* *P*<0.01; ** *P*<0.001, Mann-Whitney U-test).

Gross visual inspection of *P. gingivalis*-infected and sham-infected wild-type mice under a stereoscopic microscope (Fig. 1A–D) revealed the alveolar bone pathology. The distance between the cement-enamel junction (CEJ) and the alveolar bone crest (ABC) in the mandibular molars (measured by micro-CT) was significantly different between the infected mice and the control mice. *P. gingivalis* infection significantly increased the distance between the CEJ and the ABC in the interdental region (*P*<0.005 compared to sham-infected mice; Fig. 1E). The effect of *P. gingivalis* infection tended to be greater in B6.Apoeshl mice compared wild-type mice (*P*<0.01).

### Systemic response to oral Infection with *P. gingivalis*

**Figure 2:**
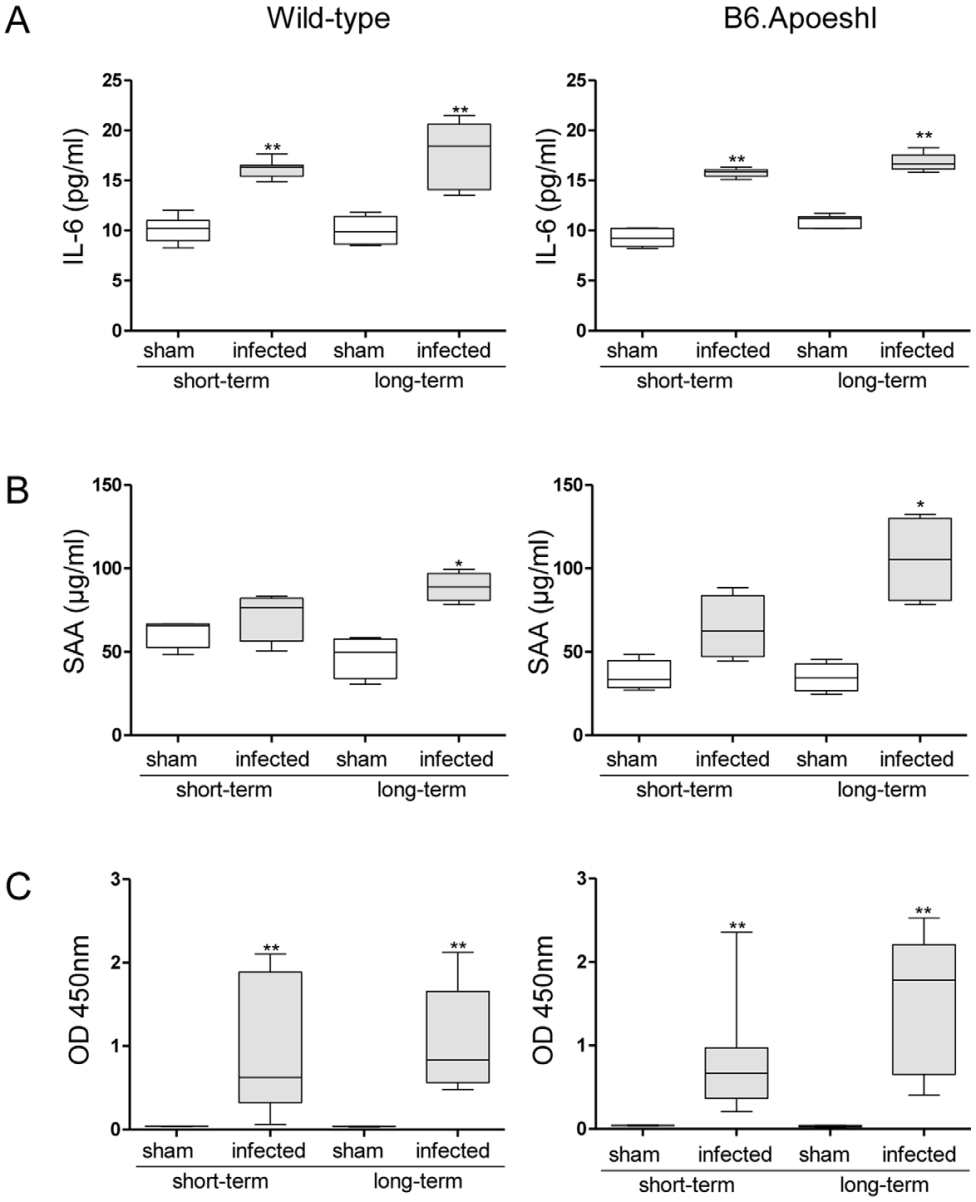
Effects of oral infection with *P. gingivalis* on serum levels of interleukin (IL)-6 (A), serum amyloid A (SAA; B), and anti-*P. gingivalis* antibody (C; N = 5 in each group). All experiments were performed in triplicate wells for each condition and repeated at least twice. Representative data are shown. Box plots present medians and the 25th and 75th percentiles as boxes and the 10th and 90th percentiles as whiskers. Significant differences were observed between the infected group and the sham-infected group (* *P*<0.05; ** *P*<0.01, Mann-Whitney U-test).

Oral infection with *P. gingivalis* induced a significant elevation in serum IL-6 levels during short-term infection, regardless of whether hyperlipidemia was present (Fig. 2A). During short-term infection, serum amyloid A (SAA) was increased in infected mice compared with those subjected to sham infection, but this difference did not reach a statistically significant level. Further elevation of IL-6 and SAA was observed during long-term infection, and significant differences were observed in both wild-type and B6.Apoeshl mice (Fig. 2B). The serum levels of *P. gingivalis* W83-specific IgG were significantly greater (*P*<0.01) in *P. gingivalis*-infected mice compared with sham-infected mice after short-term infection. In sham-infected mice, no production of antibodies against *P. gingivalis* was observed during either short-term or long-term infection periods. However, the infection did induce significantly greater antibody production during short-term infection. Further elevation of antibody levels was observed in response to long-term infection (Fig. 2C). There was no difference in antibody production observed between wild-type and B6.Apoeshl mice.

### Deterioration of the serum lipid profile id associated with the infection period

In both mouse strains, oral infection with *P. gingivalis* had no significant effect on the serum lipid profile during short-term infection. In wild-type mice, only HDL cholesterol levels were decreased by long-term infection. In contrast, all cholesterol levels shifted toward atherogenic levels during long-term *P. gingivalis* infection in B6.Apoeshl mice. Total and LDL cholesterol levels were elevated and HDL cholesterol levels were decreased in *P. gingivalis*-infected mice compared with sham-infected mice (Table 1).

**Table 1:** Serum lipid profile associated with infection period in wild-type and B6.Apoeshl mice.

Wild-type						
	Short-term			Long-term		
	Sham-infected	Infected	*P*-value	Sham-infected	Infected	*P*-value
Total	102.05±4.73	103.01±4.70	0.841	100.31±6.99	99.06±3.72	0.841
CM	0.58±0.26	0.31±0.17	0.151	0.56±0.23	0.55±0.24	0.917
VLDL	8.09±2.82	6.67±1.08	0.421	8.94±2.12	9.83±3.37	1
LDL	12.72±1.51	11.88±0.97	0.309	11.54±2.37	11.88±2.17	0.548
HDL	80.66±6.12	86.53±5.66	0.222	89.81±1.71	84.88±2.53	0.008
TG	76.79±32.59	47.37±21.04	0.151	20.43±5.21	12.41±3.05	0.016

B6.Apoeshl						
	Short-term			Long-term		
	Sham-infected	Infected	*P*-value	Sham-infected	Infected	*P*-value
Total	750.26±157.93	775.51±165.26	1	614.29±47.19	756.18±33.93	0.008
CM	43.33±5.11	35.31±2.35	0.056	41.21±3.91	35.67±2.67	0.056
VLDL	458.71±111.32	479.66±143.16	1	398.89±53.60	493.64±45.21	0.032
LDL	249.38±46.71	252.22±34.07	1	179.09±29.93	255.51±27.64	0.016
HDL	34.83±7.73	34.29±8.24	0.841	38.31±4.34	23.35±1.81	0.008
TG	45.21±41.60	59.04±26.80	0.548	67.81±8.30	38.21±10.62	0.008

Values are indicated as mg/dL.

CM indicates chylomicron; VLDL, very low density lipoprotein; LDL, low density lipoprotein; HDL, high density lipoprotein.

N = 5 in each group.

### An increase in atherosclerosis is associated with *P. gingivalis* infection in B6.Apoeshl mice but not in wild-type mice

**Figure 3:**
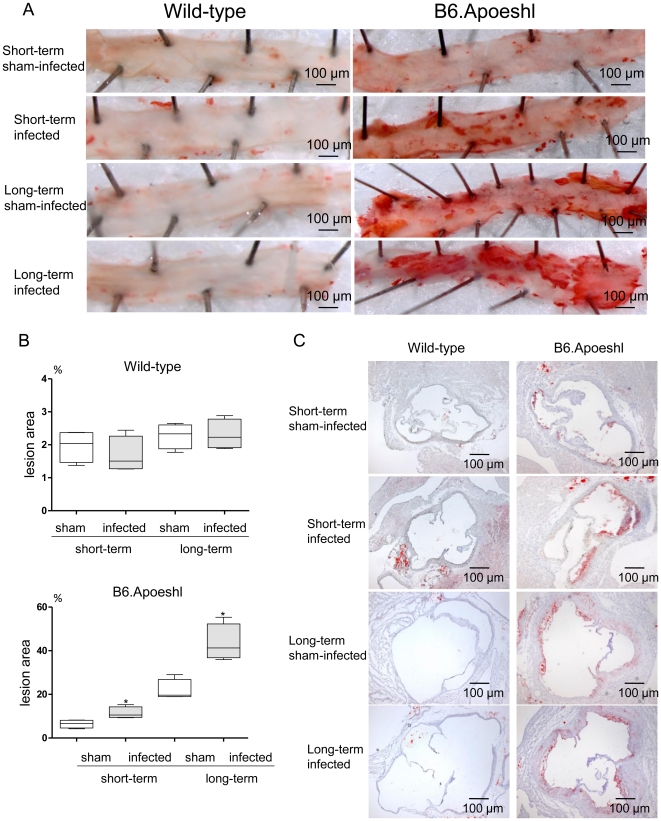
Effects of oral infection with *P. gingivalis* on aortic atherosclerosis in wild-type and B6.Apoeshl mice. (A) Representative aortas from wild-type and B6.Apoeshl mice are depicted. (B) Aortic atherosclerosis expressed as a percentage of the total area (N = 8 in each group). Box plots present medians and the 25th and 75th percentiles as boxes and the 10th and 90th percentiles as whiskers. Significant differences were observed between the infected group and the sham-infected group (* *P*<0.01, Mann-Whitney U-test). (C) Representative aortic sinus cross sections from wild-type and B6.Apoeshl mice. Original magnifications, 40×.

Analysis of aortic atherosclerosis demonstrated a significant increase in lesion area during the experimental period in the absence of infection in B6.Apoeshl mice, but not wild-type mice (short-term infection vs. long-term infection in sham-infected mice). *P. gingivalis* infection further increased the lesion size during each infection period, and atherosclerotic plaques comprised >40% of the total vessel area (Fig. 3B). A similar effect on the response to infection was observed in the aortic sinus lesion volume (Fig. 3C). Conversely, no apparent lesions were observed at any point during the experimental period in wild-type mice.

### Changes in atherogenicity-related gene expression profiles in aortic tissue are associated with the infection period

**Figure 4:**
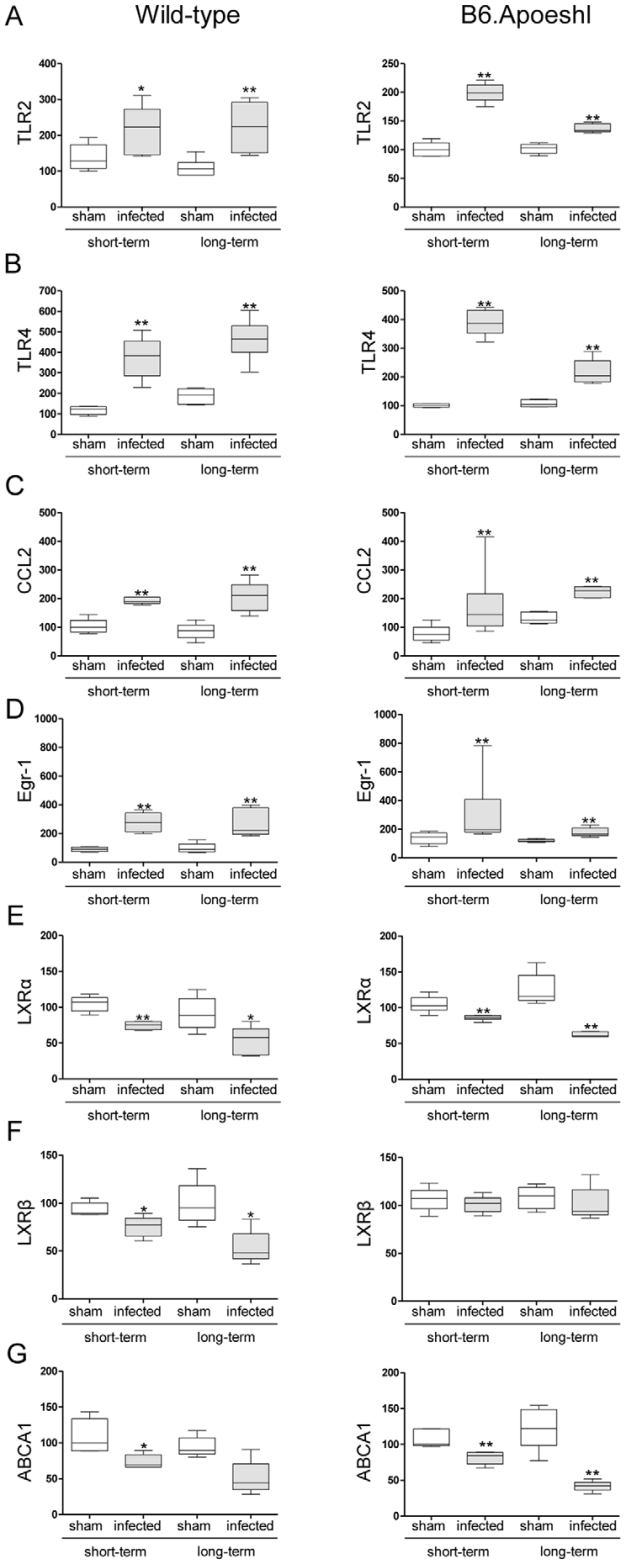
Comparison of relative gene expression levels in the aorta between the control group and the infected group or between the short-term and long-term infected groups (N = 5 in each group). The relative quantity of experimental mRNA was normalized to the relative quantity of glyceraldehyde-3-phosphate dehydrogenase (GAPDH) mRNA. The box plots present medians and the 25th and 75th percentiles as boxes and the 10th and 90th percentiles as whiskers. (* *P*<0.01, Mann-Whitney U-test)

Oral infection with *P. gingivalis* significantly increased the expression of Toll-like receptor (TLR) 2 (Fig. 4A) and TLR4 (Fig. 4B) in infected mice compared with sham-infected mice, regardless of whether hyperlipidemia was present. In wild-type mice, a longer bacterial burden induced a further elevation in gene expression, whereas the difference between sham-infected and *P. gingivalis*-infected mice was smaller in long-term infected B6.Apoeshl mice.

Chemokine (C-C motif) ligand 2 (*Ccl2*) and early growth response-1 (*Egr1*), both of which are vascular inflammation-related genes, were also significantly up-regulated in the aortic tissues of *P. gingivalis*-infected mice relative to sham-infected mice (Fig. 4C and 4D). There was no difference in the *Egr1* expression pattern either between short-term infection and long-term infection or between wild-type and B6.Apoeshl mice. The duration of the infection period did not affect *Ccl2* expression in sham-infected mice, and *P. gingivalis* infection consistently induced the up-regulation of gene expression.

Because of the importance of lipid metabolism in atherogenesis and the involvement of infection and inflammation in lipid metabolism, the gene expression levels of several cholesterol transport molecules liver X receptors (LXR) α, βand ATP-binding cassette A1 (ABCA1) were examined. Both *Lxrα* and *Lxrβ* were down-regulated during short-term infection, and further down-regulation was observed during long-term infection in wild-type mice (Figs. 4E and 4F). In B6.Apoeshl mice, *Lxrα* showed changes similar to those in wild-type mice, whereas *Lxrβ* did not show any difference following infection. *Abca1* was down-regulated in *P. gingivalis*-infected mice, with a greater reduction being observed during long-term infection. There was no difference in *Abca1* expression between wild-type and B6.Apoeshl mice (Fig. 4G).

### Changes in atherogenicity-related gene expression profiles in the liver are associated with the infection period

**Figure 5:**
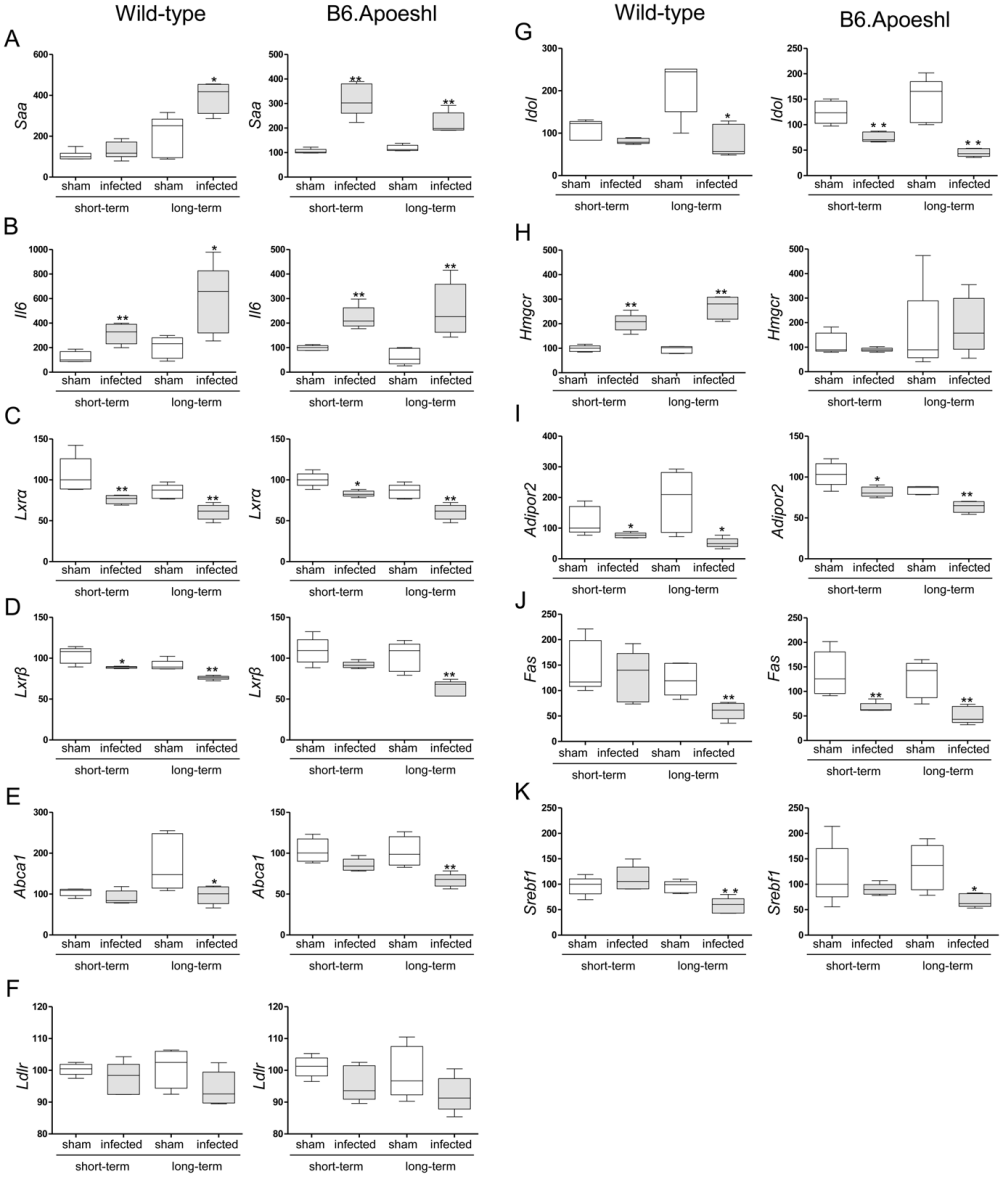
Comparison of the relative gene expression levels in the liver between the control group and the infected groups or between the short-term and long-term infected groups (N = 5 in each group). The relative quantity of mRNA was normalized to the relative quantity of GAPDH mRNA. The box plots present medians and the 25th and 75th percentiles as boxes and the 10th and 90th percentiles as whiskers. (* *P*<0.05; ** *P*<0.01, Mann-Whitney U-test)

The expression levels of the inflammation-related markers *Saa1/2* and *Il6* were significantly up-regulated during *P. gingivalis* infection (Figs. 5A and 5B). The expression pattern showed differences between wild-type and B6.Apoeshl mice. Long-term infection tended to produce enhanced up-regulation of *Saa* in wild-type mice, whereas the difference in gene expression was smaller between sham-infected and *P. gingivalis*-infected B6.Apoeshl mice during long-term infection. No such difference was observed for *Il6* expression. The lipid metabolism-related genes *Lxrα* and *Lxrβ*were down-regulated during short-term infection and further down-regulated under long-term infection, regardless of whether hyperlipidemia was present (Figs. 5C and 5D). The gene expression profile of *Abca1* showed a trend similar to that of the *Lxr*s, but a significant reduction was only observed during long-term infection (Fig. 5E). In addition, LDL receptor (*Ldlr*) and inducible degrader of the LDL receptor (*Idol*), another LXR target gene, were analyzed. The expression of *Ldlr* tended to be lower in *P. gingivalis*-infected mice compared with sham-infected mice (Fig. 5F). Consistent with *Lxrs* expressions, *Idol* expression was also decreased in *P. gingivalis*-infected mice compared with sham-infected mice. The change was greater during long-term infection in both wild-type and B6.Apoeshl mice (Fig. 5G).

In contrast to the *Lxr*s and *Abca1*, a marked difference in hydroxymethylglutaryl-coenzyme A reductase (*Hmgcr*) expression was observed between wild-type and B6.Apoeshl mice. Infection with *P. gingivalis* significantly increased the expression of *Hmgcr* as the bacterial burden in wild-type mice increased, while there was no change in the expression levels of this gene in B6.Apoeshl mice (Fig. 5H). A significant down-regulation was also observed in *Adipor2*, which is another antiatherogenic molecule (Fig. 5I).

Because triglyceride levels were significantly down-regulated during *P. gingivalis* infection, particularly during long-term infection, the expression levels of genes critical for the regulation of triglyceride synthesis were analyzed. Both fatty acid synthase (*Fas*) and sterol regulatory element-binding protein 1c (*Srebf1* in mice) expression levels were significantly down-regulated during long-term infection but not during short-term infection in wild-type mice (Figs. 5J and 5K). In B6.Apoeshl mice, *Fas* expression was significantly lower in infected mice compared to sham-infected mice, regardless of the length of the infection period. *Srebf1* expression levels were significantly down-regulated only during long-term infection in this strain of mice.

### TLR ligands induce phosphorylation of IRF3 in macrophages

**Figure 6:**
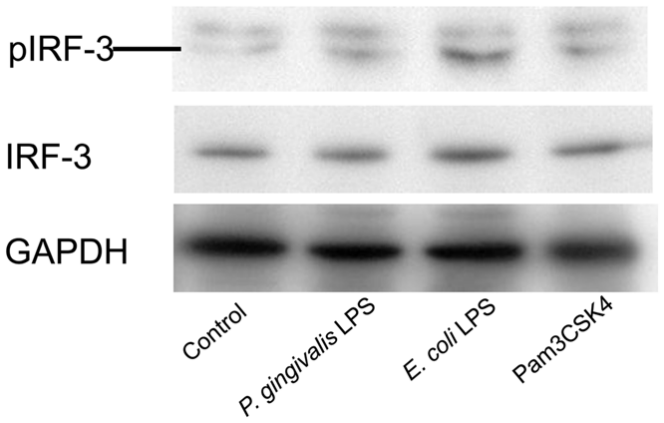
Induction of IRF3 phosphorylation in RAW 264.7 macrophages by TLR2 and TLR4 ligands. Cells were stimulated with 1.0 µg/ml of *P. gingivalis* LPS, 0.1 µg/ml of *E. coli* LPS or 0.1 µg/ml of Pam3CSK4 for 12 hrs. Phosphorylation of IRF3 cellular extracts was analyzed by western blotting. Blot is a representative of three independent experiments.

As shown in Fig. 6, the TLR4 ligand *E. coli* LPS and TLR2 ligand Pam3C SK4 clearly induced the phosphorylation of IRF3 in macrophages. *P. gingivalis* LPS, which is known to act as an agonist for both TLR2 and TLR4, also induced IRF3 phosphorylation, although the activity of *P. gingivalis* LPS was much less potent than that of *E. coli* LPS.

### Effects of *P. gingivalis* and *E. coli* LPS on cholesterol uptake and efflux from macrophages

**Figure 7:**
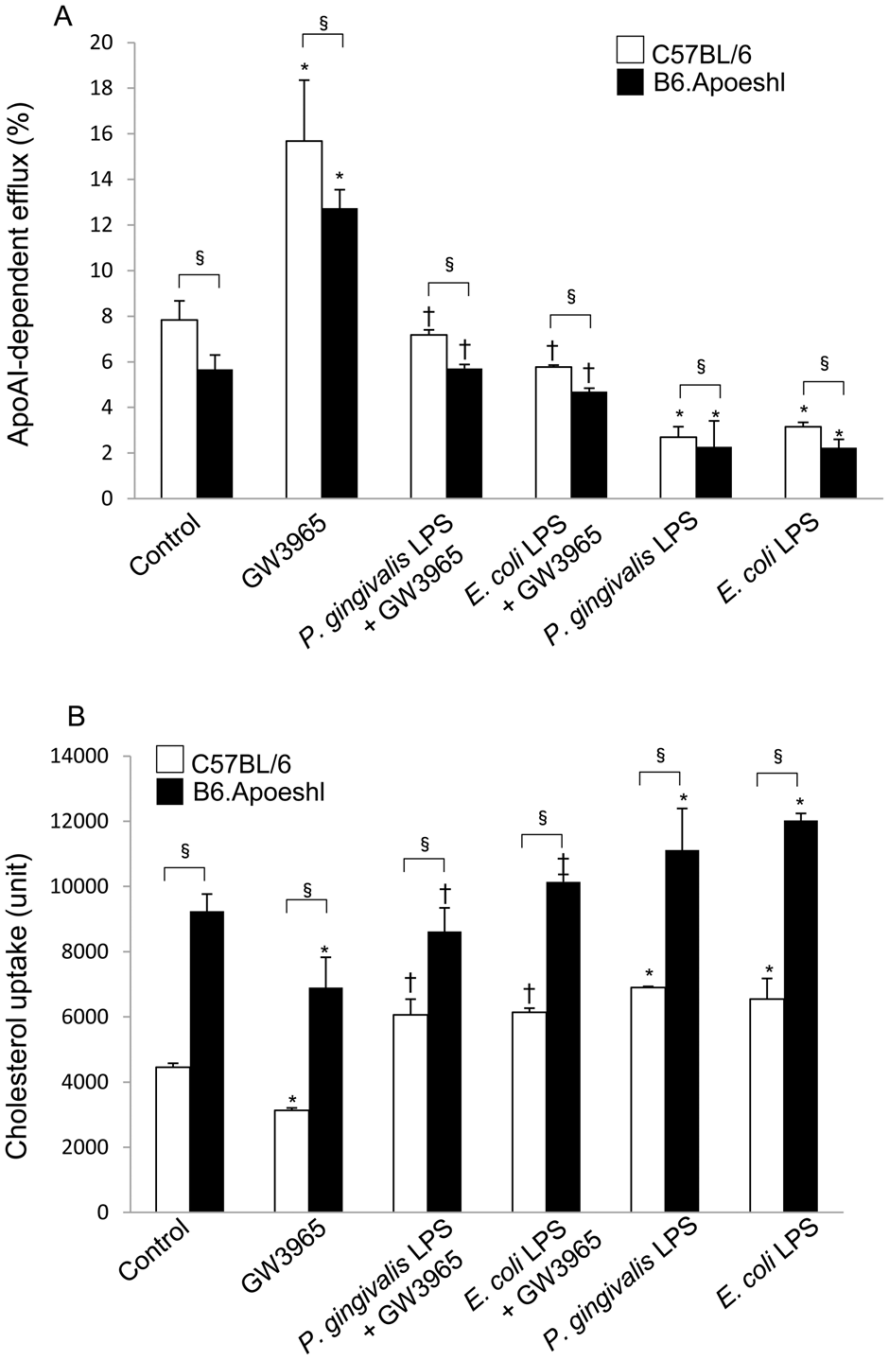
Lipopolysaccharide from *P. gingivalis* and *E. coli* inhibit cholesterol efflux and enhance cholesterol uptake. (A) Peritoneal macrophages from wild-type and B6.Apoeshl mice were loaded with BODIPY-cholesterol and treated with vehicle, 1 µg/ml *P. gingivalis* LPS or 0.1 µg/ml *E. coli* LPS in the presence or absence of 1 µM GW3965. Data are presented as apoA1-dependent cholesterol efflux. (B) Cholesterol uptake was estimated as the total of cellular and effluxed cholesterol. The results are shown as the mean **±** S.D. of three independent experiments. There were significant differences found between the wild-type and B6.Apoeshl macrophages (unpaired t-test; § *P*<0.05) and between control and LPS-treated macrophages in the absence (paired t-test; * *P*<0.05) or presence of GW3965 (paired *t*-test; † *P*<0.05).

Oral infection with *P. gingivalis* down-regulated the expression of the *Lxr*s and *Abca1* in both strains of mice. However, in contrast to the progression of the atherosclerotic lesions in B6.Apoeshl mice, no development of such lesions was observed in wild-type mice. These results suggested that either cholesterol efflux may be much more impaired by the infection in B6.Apoeshl mice than wild-type mice. Alternatively cholesterol efflux may be similar between wild-type and B6.Apoeshl mice, but cholesterol uptake may be higher in B6.Apoeshl mice. This would result in increased cholesterol accumulation and lesion progression. To distinguish between these two hypotheses, the effect of LPS treatment on cholesterol efflux by peritoneal macrophages was quantified in both strains of mice. We found that the apoA1-dependent cholesterol efflux induced by GW3965, a synthetic LXR agonist, was significantly inhibited by LPS treatment. The effect of GW3965 and inhibitory effect of LPS on GW3965-induced apoA1-dependent cholesterol efflux were similar between wild-type mice and B6.Apoeshl mice (Fig. 7A). Basal cholesterol uptake of B6.Apoeshl macrophages was much higher compared to that of wild-type macrophages. LPS treatment in the absence of GW3965 significantly increased cholesterol uptake in both wild-type macrophage and B6.Apoeshl macrophage (Fig. 7B). These results suggested that increased atherosclerotic lesions may be due to the increased uptake of cholesterol into B6.Aoeshl macrophages compared with wild-type macrophages, rather than differences in cholesterol efflux. The differences of these cellular characteristics were further manifested by the LPS stimulation.

### Effects of *P. gingivalis* and *E. coli* LPS on LDLR expression in macrophages

**Figure 8:**
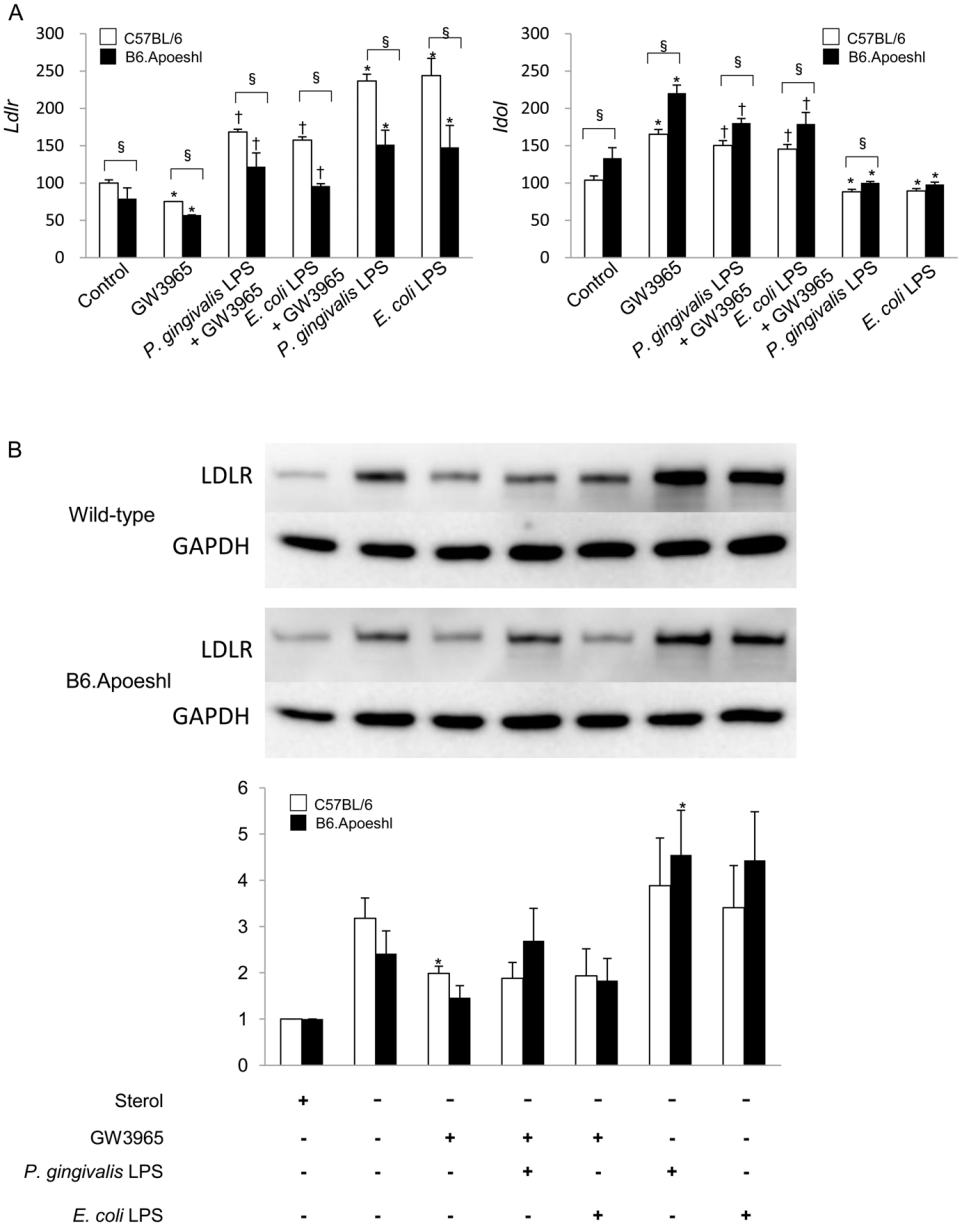
Lipopolysaccharide from *P. gingivalis* and *E. coli* alter the LDLR expression in macrophages. Peritoneal macrophages from wild-type and B6.Apoeshl mice were cultured in the sterol depletion medium for 8 hours and the effect of LPS was evaluated in the presence or absence of GW3965 following 18 hours of incubation. (A) Total RNA was extracted from the cells and gene expression of LDLR and Idol was analyzed by real-time PCR. Data are expressed as mRNA expression relative to the expression without stimulation. Results are shown as mean ± S.D. of three independent experiments. There were significant differences between the wild-type and B6.Apoeshl macrophages (unpaired t-test; § *P*<0.05) and between control and LPS treated macrophages in the presence (paired t-test; † *P*<0.01) or absence of GW3965 (paired *t*-test; * *P*<0.05). (B) Cell lysates were separated by SDS-PAGE and immunoblotted with anti-LDLR and anti-GAPDH antibodies. Results are representative of those in three independent experiments. The result of densitometric analysis of western blotting shown as mean ± S. E. of three independent experiments. Significant difference between sterol-depletion control and the different LPS stimulations are indicated (paired *t*-test; * *P*<0.05).

The gene expression of LDLR in B6.Apoeshl macrophages was lower than that in wild-type macrophages and stimulation with *P. gingivalis* LPS and *E. coli* LPS increased the gene expression in both strains of mice. Although GW3965 suppressed the *Ldlr* gene expression, either LPS abrogated the effect of GW3965. In contrast to the *Ldlr*, the gene expression of *Idol* was higher in B6.Apoeshl macrophages compared with wild-type macrophages. While GW3965 increased the Idol expression in macrophages of both strains of mice, LPS suppressed the effect of GW3965 (Fig. 8A).

Culture in the sterol-depletion medium strongly induced LDLR protein expression in the macrophage and the addition of GW3965 potently decreased LDLR protein levels in macrophages. P. gingivalis LPS stimulation not only markedly attenuated the suppressive activity of GW3965 on the LDLR expression but also up-regulated the LDLR protein expression in B6.Apoeshl mice. Both LPS enhanced LDLR expression in the absence of GW3965 (Fig. 8B).

## Discussion

In both wild-type and B6.Apoeshl mice, oral infection with *P. gingivalis* induced alveolar bone resorption, which is a characteristic feature of chronic periodontitis. Both strains of mice also showed elevated levels of serum antibodies against this bacterium, indicating a systemic immune response. Because periodontal tissue destruction is stimulated by inflammatory mediators, rather than by the bacteria themselves [16], the alterations in gene expression levels, the serum lipid profile and serum inflammatory mediators observed in this study could be a consequence of oral infection with *P. gingivalis*. Despite the various systemic effects of oral infection in wild-type mice, the infection did not induce the development of atherosclerotic lesions. The gene expression profiles in the aorta and liver were consistent with proatherogenic profiles. These results are in agreement with the findings related to the effects of other infectious agents, such as *C. pneumonia*, on atherogenesis in mice. However, *C. pneumonia* inoculation in wild-type mice fed a high-fat diet accelerated hypercholesterolemia-induced atherosclerosis [17]. This suggests that infection may play a synergistic role with other preexisting factors, such as hyperlipidemia, to result in the development of atherosclerosis. Therefore, it is important to clarify whether *P. gingivalis* infection has the same effects as *C. pneumonia*. In this context, previous studies have examined the role of periodontal infection using ApoE-deficient mice [9], [10]. These studies demonstrated that either oral or intravenous *P. gingivalis* infection accelerated atherosclerosis and raised the levels of systemic inflammatory markers. However, the infection period in these studies was relatively short (3 weeks), and there was no information provided about whether long-term infection mimicked human chronic periodontitis. Furthermore, the ways in which oral infection affects the function of the aorta and liver, which are the organs most relevant to atherogenesis, are unknown.

Our analysis of gene expression levels in the aorta provided new insights into both the mechanisms linking periodontal infection with atherosclerosis and the role of hyperlipidemia in the immune response of B6.Apoeshl mice to *P. gingivalis* infection. During short-term infection, aortic expression of both *Tlr2* and *Tlr4* was significantly up-regulated. However, the stimulatory effect of *P. gingivalis* oral infection was attenuated during long-term infection. Because such attenuation is not seen in wild-type mice, in which the expression of *Tlr2* and *Tlr4* was further elevated after long-term infection compared with short-term infection, the deletion of the *ApoE* gene and/or the resulting hyperlipidemia may have affected the endothelial response. Likewise, the expression of *Egr1* and *Ccl2* was significantly enhanced in B6.Apoeshl mice but this effect was less pronounced than in wild-type mice. Because the expression of both *Egr1* and *Ccl2* is positively regulated by IL-6 in endothelial cells [18], increased serum IL-6 levels in B6.Apoeshl mice compared with wild-type mice would be expected to enhance the transcription of these molecules. Contrary to this assumption, IL-6 did not up-regulate these molecules. These results again suggest that abnormal lipid metabolism may have affected inflammatory responses in endothelial cells. The lipid metabolism–inflammatory response relationship was further highlighted by the observation of alveolar bone resorption. The effect of *P. gingivalis* infection tended to be greater in B6.Apoeshl mice than in wild-type mice. Further studies should be directed at understanding the role of lipid metabolism during the inflammatory response in blood vessels.

Additionally, infection and subsequent inflammation affected lipid metabolism-related gene expression. While the observed changes in *Lxrα* and *Abca1* expression were similar between wild-type and B6.Apoeshl mice with both short-term and long-term infections, *Lxrβ* gene expression was not affected by oral infection with *P. gingivalis* in B6.Apoeshl mice.

LXRs have been identified as central regulators of lipid homeostasis. LXRα is expressed primarily in the liver, intestine, adipose tissue, and macrophages, whereas LXRβ is expressed in many cell types. Endothelial cells also express functional LXRs, and treatment with LXR agonists can up-regulate ABC transporters. This result suggests that LXRs have an antiatherogenic effect in endothelial cells [19], [20]. A number of studies have demonstrated that ABCA1 is an efficient exporter of cholesterol from macrophages and other cells, a major determinant of plasma HDL cholesterol levels and a potent cardioprotective factor [21]. The concomitant reduction of *Lxrα* and *Abca1* seen in the livers of infected mice suggests that LXRs are functionally affected by oral infection with *P. gingivalis*, possibly via crosstalk between LXR and TLR signaling. Castillo et al. [22] demonstrated that activation of TLR3 and TLR4 by microbial ligands blocks the induction of LXR target genes, including *Abca1*, in cultured macrophages and aortic tissues *in vivo*. Crosstalk between LXR and TLR signaling is mediated by interferon regulatory factor 3 (IRF3), a specific effector of TLR3/4 that inhibits the transcriptional activity of LXR. *P. gingivalis* contains diverse TLR ligands, including LPS, lipoproteins, and CpG DNA, which are recognized by TLR4, TLR2, and TLR9, respectively. Although the agonistic activity of *P. gingivalis* LPS is weaker than *E. coli* LPS [23], we found that *P. gingivalis* LPS could also induce phosphorylation of IRF3 in macrophages, albeit to a lesser extent than *E. coli* LPS (Fig. 6). Therefore, TLR signaling-mediated IRF3 activation may be a mechanism for the down-regulation of *Lxr* and *Abca1*.

The functional effect of the change in *Lxr*s and *Abca1* expression levels on the shift in serum lipoproteins (particularly HDL cholesterol) was confirmed by the inhibition of cholesterol efflux by *P. gingivalis* LPS and *E. coli* LPS in peritoneal macrophages. GW3965-induced apoA1-dependent cholesterol efflux was completely abolished by stimulation with LPS. However, the rate of basal and GW3965-induced cholesterol efflux was lower in B6.Apoeshl mice compared with wild-type mice. On the other hand, cholesterol uptake was higher in B6.Apoeshl mice compared to wild-type mice and the uptake was enhanced by the LPS stimulation in both strains of mice.

Plasma cholesterol level is regulated by the LDLR and the expression of the LDLR is regulated at transcriptional and post translational levels. Recently, Zelcer et al. [24] found that LDLR expression is controlled by the LXR-Idol axis where LXR induces Idol, an E3 ubiquitin ligase that triggers LDLR degradation. Given that *Lxr*s are down-regulated in the *P. gingivalis*-infected mice, it is probable that Idol expression is also suppressed in the infected mice compared to sham-infected mice and this change may relate to up-regulation of LDLR. In fact, the expression of the *Idol* gene was significantly lower in the liver of the infected mice and the change was more prominent during the long-term infection in B6.Apoeshl mice. Although the *Ldlr* expression tended to be lower in *P. gingivalis*-infected mice compared to sham-infected mice, the difference was not statistically significant. These changes may have negative effect on Idol-mediated LDLR degradation resulting in the enhanced LDLR expression and subsequent decrease of plasma cholesterol. However, total and LDL cholesterols showed no change in wild-type mice but rather elevation in B6.Apoeshl mice during the long-term infection. *In vitro* stimulation of macrophages with LPS attenuated the GW3965-mediated suppression of the LDLR expression and the effect of *P. gingivalis* LPS was more potent than *E. coli* LPS. The reason for this paradox is unclear. However, Li et al. [25] showed that the acute inflammatory response decrease LDL receptor expression in the liver. In contrast, the same inflammatory stimuli increased the LDLR expression in macrophages. Our results are consistent with these findings. In addition to Idol, the proprotein convertase subtilisin-like kexin type 9 (PCSK9) is also implicated in the regulation of LDLR expression [26]. Although we have not determined whether PCSK9 contributes significantly to LDLR degradation and how its activity is affected by LPS, Feingold et al. [27] showed that inflammation stimulates PCSK9 expression leading to increased LDLR degradation but the underlying mechanisms remain elusive. Nevertheless, oral infection of *P. gingivalis* does affect the expression of the genes that regulate LDLR expression at protein level.

In wild-type mice, genes involved in lipid metabolism, but not the lipid profile itself, were found to be altered by short-term infection with *P. gingivalis*. By contrast, long-term infection reduced HDL cholesterol levels. The exact reasons for the discrepancy between gene expression levels and the lipid profile are not known. However, because the reduction in the expression of the *Abca1* gene, a rate-limiting factor in HDL biogenesis [28], was more pronounced during long-term infection than during short-term infection, it is possible that the suppression of *Abca1* during long-term infection was sufficiently robust to induce a decrease in HDL cholesterol. Few effects of short-term infection with *P. gingivalis* on total cholesterol and the size of the lipoproteins in plasma have been reported [10]. Therefore, it appears that longer-term and chronic infections are necessary to shift the plasma lipid profile toward an atherogenic state, despite the observation of proatherogenic changes in gene expression during short-term infection.

In addition to its effects on HDL cholesterol, *P. gingivalis* infection induced a significant down-regulation of serum triglycerides during long-term infection. *Fas* and *Srebf1*, which are genes involved in the regulation of triglyceride synthesis, were also down-regulated. Enzymes involved in triglyceride synthesis are transcriptionally regulated by SREBP-1c [29] and LXRs [30] in the liver. LXRs are also important regulators of FAS [31]. The conversion of glucose into triglycerides is nutritionally regulated, and both insulin and glucose signaling pathways are involved. Given that LXRs are key regulators of insulin and glucose signaling pathways that were down-regulated in infected mice of both strains, the resulting down-regulation of *Fas* and *Srebf1* and subsequent decrease in serum triglycerides could be due to *P. gingivalis* infection.

Severe periodontitis has been associated with a modest decrease in HDL and LDL cholesterol levels and a more robust increase in plasma triglycerides [32], [33]. Although the effects of periodontal infection on the lipid profile of long-term infected B6.Apoeshl mice are slightly different from those seen in human periodontitis, particularly with regard to triglycerides, it is clear that infection does affect lipid metabolism. However, further studies are necessary to clarify the relationship between chronic infection and lipid metabolism. Nevertheless, the systemic inflammation induced by *P. gingivalis* oral infection is associated with a shift to a proatherogenic serum lipid profile that leads to elevated LDL cholesterol and decreased HDL cholesterol levels [34].

In summary, *P. gingivalis* is a representative periodontopathic bacterium that does not have the ability to induce atherosclerosis but can accelerate the progression of atherosclerosis by affecting blood vessel and liver function when hyperlipidemia is present. The results of the present study provide a potential mechanism for this bacterium's contribution to atherogenesis and shed light on the underlying mechanism by which periodontitis affects atherosclerosis and subsequent CHD in humans.

## Materials and Methods

### Reagents

GW3965 hydrochloride, Sandoz 58-035, Apolipoprotein A-1, simvastatin sodium salt, mevalonic acid and lipoprotein-deficient fetal bovine serum (LPDS) were purchased from Sigma-Aldrich (St. Louis, MO). BODIPY-cholesterol (23-(dipyrrometheneboron difluoride)-24-norcholesterol was obtained from Avanti Polar Lipids, Inc. (Alabaster, AL). M-PER Mammalian Protein Extraction Reagent and Pierce BCA Protein Assay kit were obtained from Thermo Scientific (Rockford, IL). Pam3Cys-SKKKK x 3HCl (Pam3CSK4) was purchased from EMC microcollections (Tuebingen, Germany). Lipopolysaccharide (LPS) from *Escherichia coli* O111:B4 was purchased from Sigma. LPS from *P. gingivalis* 381 was kindly provided by H. Kumada and T. Umemoto (Department of Microbiology, Kanagawa Dental University, Yokosuka, Japan). Rabbit anti-mouse IRF3 (Santa Cruz Biotechnology, Santa Cruz, CA), rabbit anti-mouse phospho-IRF3 (Cell Signaling Technology, Danvers, MA), rabbit anti-mouse GAPDH (Santa Cruz Biotechnology), peroxidase labeled anti-rabbit antibody (GE Healthcare, Munich, Germany) and ECL Plus Western Blotting Reagent Pack (Amersham Biosciences, Buckinghamshire, UK) were used for western blotting experiments.

### Mice

All experiments were performed in accordance with the Regulations and Guidelines on Scientific and Ethical Care and Use of Laboratory Animals of the Science Council of Japan, enforced on June 1, 2006, and approved by the Institutional Animal Care and Use Committee at Niigata University (permit number 39). Six-week-old male C57BL/6 mice and spontaneously hyperlipidemic male C57BL/6.KOR -*Apoe^shl^* (B6.Apoeshl) mice [35], [36] were obtained from Japan SLC, Inc. (Shizuoka, Japan). The mice were maintained under specific pathogen-free conditions and fed regular chow and sterile water until the commencement of infection at 8 weeks of age.

### Bacterial Cultures

*P. gingivalis* strain W83 was cultured in modified Gifu anaerobic medium (GAM) broth (Nissui, Tokyo, Japan) in an anaerobic jar (Becton Dickinson Microbiology System, Cockeysville, MD) in the presence of an AnaeroPack™ (Mitsubishi Gas Chemical Co. Inc., Tokyo, Japan) for 48 hours at 37°C. Bacterial suspensions were prepared in phosphate-buffered saline (PBS) without Mg^2+^/Ca^2+^ using established growth curves and spectrophotometric analysis. The number of CFUs was standardized by measuring optical density (600 nm).

### Oral Infection

The murine experimental periodontitis model was developed according to Baker et al. [37] with slight modifications. The animals received sulfamethoxazole and trimethoprim at final concentrations of 700 µg/ml and 400 µg/ml, respectively, in water bottles *ad libitum* for 10 days. This treatment was followed by 3 days without antibiotics. The experimental group was then infected. A total of 10^9^ CFU of live *P. gingivalis* suspended in 100 µl of PBS with 2% carboxymethyl cellulose (Sigma-Aldrich) was given to each mouse via a feeding needle. This suspension was given either 10 times (short-term infection) or 42 times (long-term infection) at 3-day intervals. The control group received the same pretreatment and was sham infected without the *P. gingivalis*. Two days after the final treatment, the mice were fasted for 16 hours, and oral swabs were obtained and tested for the presence of *P. gingivalis* as previously described [38]. Mice were then euthanized with CO2, and their tissues were removed.

### Quantification of Alveolar Bone Loss

For quantitative three-dimensional (3-D) analysis of alveolar bone loss, mandibles were scanned by micro-computerized tomography (micro-CT) (Skyscan 1174; Skyscan, Kontich, Belgium). The micro-CT settings were as follows: pixel size, 1024×1024; slice thickness, 6 µm; magnification, 6×; voltage, 50 kV; and electrical current, 0.08 mA. The 3-D views were constructed with the imaging software program CTan Ver.1.5.0 (SkyScan), and alveolar bone loss was analyzed with the software program CTvol ver.1.9.4 (SkyScan). The sagittal plane of the specimens was set parallel to the x-ray beam axis. The distance from the distal CEJ of the first molar in the middle of the lingual-distal root to the interdental ABC between the first and second molars was measured in the sagittal plane parallel to the lingual-distal root. The measurements of alveolar bone loss in mice were performed by blinded examiners (SM and DY).

### Serum Lipoprotein, SAA, IL-6 and IgG Antibody Levels

Sera were obtained prior to euthanasia, and serum cholesterol and triglyceride profiles were analyzed at Skylight Biotech Inc. (Akita, Japan). Serum IL-6 and serum amyloid A (SAA) were measured using commercial ELISA kits (Thermo Scientific and Kamiya Biomedical, Seattle, WA, respectively). *P. gingivalis*-specific IgG antibodies levels were measured by ELISA according to a previously described method [33].

### Real-time PCR

Total RNA isolated from aorta, liver tissues and macrophages was extracted using an RNeasy Mini kit and treated with DNase I (Qiagen, Germantown, MD) according to the manufacturer's instructions. Aliquots of RNA were then reverse-transcribed to cDNA using random primers (Takara Bio Inc., Shiga, Japan) and M-MLV reverse transcriptase (Invitrogen, Carlsbad, CA). Primers and probes specific for real-time PCR were purchased from Applied Biosystems (Foster City, CA). Reactions were carried out in a 25- µl mixture in the ABI PRISM 7900HT Sequence Detection System (Applied Biosystems) using TaqMan Gene Expression Assays (Applied Biosystems) containing 900 nM primer and 250 nM probe. The reactions consisted of a 10-minute incubation at 95°C, followed by 40 cycles of a two-step amplification procedure consisting of annealing/extension at 60°C for 1 minute and denaturation for 15 seconds at 95°C. ABI PRISM SDS 2.0 software (Applied Biosystems) was used to analyze the standards and carry out the quantifications. The relative quantity of each mRNA was normalized to the relative quantity of glyceraldehyde-3-phosphate dehydrogenase (GAPDH) mRNA.

### Assessment of the Atherosclerotic Lesion Area

The extent of atherosclerotic lesions in the aortic tree was determined by en face quantification. The aorta was harvested, opened longitudinally, fixed with 4% PFA/PBS and stained with oil red O (Sigma-Aldrich). The stained area was analyzed using ImageJ 1.42 q (Wayne Rasband, National Institutes of Health) and expressed as a proportion of the lesion within the total vessel area. In addition, frozen sections of the proximal aorta were prepared [39]. Briefly, five sections (10 µm thick) per mouse, each separated by 100 µm, were stained with oil red O.

### Western blots

For IRF-3 analysis, RAW 264.7 macrophages were seeded in a 24-well culture plate (TPP, Trasadingen, Switzerland) at a concentration of 1×10^6^ cells/ml in D-MEM supplemented with 1% penicillin/streptomycin. After 12 hours of incubation, the cells were stimulated for 30 minutes with 1 µg/ml of *P. gingivalis* LPS, 0.1 µg/ml of *E. coli* LPS or 0.1 µg/ml of Pam3CSK4 in medium.

The protein expression of LDLR was evaluated in peritoneal macrophages obtained from wild-type and B6.Apeshl mice. The macrophages were collected 4 days after intraperitoneal injection of aged sterile 3% thioglycollate and cultured in a 12-well culture plate (TPP, Trasadingen, Switzerland) at a concentration of 1×10^6^/well in D-MEM supplemented with 10% LPDS, 5 µM simvastatin, and 100 µM mevalonic acid (sterol-depletion medium) for 8 hours and then treated with LPS from *P. gingivalis* (1 µg/ml) and *E. coli* (0.1 µg/ml) in the presence or absence of GW3965 (1 µM) for 18 hours.

The cultured cells were washed twice with ice-cold PBS, and the protein was extracted using M-PER Mammalian Protein Extraction Reagent (Pierce Biotechnology) supplemented with a Halt Protease Inhibitor Cocktail Kit (Thermo Scientific) and Halt Phosphatase Inhibitor Cocktail (Thermo Scientific) according to the manufacturer's instructions. Cell debris was pelleted by centrifugation at 12000 x g for 10 minutes at 4°C. The protein concentration in the supernatant was determined using a Pierce BCA Protein Assay kit (Thermo Scientific) according to the manufacturer's instructions.

Twelve micrograms of each sample was solubilized using SDS sample buffer, separated by SDS-PAGE, transferred to a polyvinylidene difluoride membrane (Immobilon-P; Millipore Co., Bedford, MA), subjected to western blotting with each antibody, and developed with ECL using Lumi Vision PRO 400EX (Aisin, Aichi, Japan). For reprobing of GAPDH, the membranes were washed 3 times with wash buffer (Tris-buffered saline containing 0.1% Tween-20 and 0.5% BSA) and subjected to western blotting with anti-GAPDH antibody, as described above.

### Cholesterol influx and efflux in macrophages

Peritoneal macrophages from wild-type and B6.Apoeshl mice were collected 4 days after intraperitoneal injection of aged sterile 3% thioglycollate and plated in a 12-well culture plate (TPP, Trasadingen, Switzerland) at a concentration of 1×10^6^/well in D-MEM containing 5% FBS and 1% penicillin/streptomycin, hereafter referred to as medium. Efflux assays were slightly modified from those described by Venkateswaran et al. [40]. Adherent cells were incubated with medium supplemented with ACAT inhibitor (58-035; 2 µg/ml) and BODIPY-cholesterol (10 µg/ml) for 24 hrs. The labeled cells were then treated with *P. gingivalis* LPS (1.0 µg/ml) or *E. coli* LPS (0.1 µg/ml) and/or GW3965 for 4 hrs. After cells were washed with HBSS, ApoA1 (10 µg/ml) was added to the culture and the cells were incubated for and additional12 hrs. Following incubation, the supernatants were removed and the cells were dissolved using M-PER Mammalian Protein Extraction Reagent. The labeled cholesterol in the supernatants and cell fractions was determined using a Versa Fluor Fluorometer (Bio-Rad, Hercules, CA) equipped with the appropriate filters. Uptake of cholesterol in the cells was estimated as the total of released and cellular labeled cholesterol, whereas the ApoA1-dependent efflux of labeled cholesterol from the cells into the medium was determined by calculating the percentage of the total labeled cholesterol in the cells for each condition.

### Statistical Analysis

Nonparametric data were evaluated using the Mann-Whitney U test with GraphPad Prism (GraphPad Software, Inc., La Jolla, CA) for two-group comparisons. A probability value (*P*) <0.05 was considered statistically significant.

## References

[1] O'Connor S, Taylor C, Campbell LA, Epstein S, Libby P (2001). Potential infectious etiologies of atherosclerosis: a multifactorial perspective.. Emerg Infect Dis.

[2] Chiu B, Viira E, Tucker W, Fong IW (1997). *Chlamydia pneumoniae*, cytomegalovirus, and herpes simplex virus in atherosclerosis of carotid artery.. Circulation.

[3] Mendall MA, Goggin PM, Molineaux N, Levy J, Toosy T (1994). Relation of *Helicobacter pylori* infection and coronary heart disease.. Br Heart J.

[4] Bahekar AA, Singh S, Saha S, Molnar J, Arora R (2007). The prevalence and incidence of coronary heart disease is significantly increased in periodontitis: a meta-analysis.. Am Heart J.

[5] Humphrey LL, Fu R, Buckley DI, Freeman M, Helfand M (2008). Periodontal disease and coronary heart disease incidence: a systematic review and meta-analysis.. J Gen Intern Med.

[6] Epstein SE, Zhou YF, Zhu J (1999). Infection and atherosclerosis: emerging mechanistic paradigms.. Circulation.

[7] Buhlin K, Hultin M, Norderyd O, Persson L, Pockley AG (2009). Periodontal treatment influences risk markers for atherosclerosis in patients with severe periodontitis.. Atherosclerosis.

[8] Nakajima T, Honda T, Domon H, Okui T, Kajita K (2010). Periodontitis-associated up-regulation of systemic inflammatory mediator level may increase the risk of coronary heart disease.. J Periodont Res.

[9] Gibson FC, Hong C, Chou HH, Yumoto H, Chen J (2004). Innate immune recognition of invasive bacteria accelerates atherosclerosis in apolipoprotein E-deficient mice.. Circulation.

[10] Lalla E, Lamster IB, Hofmann MA, Bucciarelli L, Jerud AP (2003). Oral infection with a periodontal pathogen accelerates early atherosclerosis in apolipoprotein E-null mice.. Arterioscler Thromb Vasc Biol.

[11] Li L, Messas E, Batista EL, Levine RA, Amar S (2002). *Porphyromonas gingivalis* infection accelerates the progression of atherosclerosis in a heterozygous apolipoprotein E-deficient murine model.. Circulation.

[12] Wilson W, Taubert KA, Gewitz M, Lockhart PB, Baddour LM (2007). Prevention of infective endocarditis: guidelines from the American Heart Association: a guideline from the American Heart Association Rheumatic Fever, Endocarditis, and Kawasaki Disease Committee, Council on Cardiovascular Disease in the Young, and the Council on Clinical Cardiology, Council on Cardiovascular Surgery and Anesthesia, and the Quality of Care and Outcomes Research Interdisciplinary Working Group.. Circulation.

[13] D'Aiuto F, Parkar M, Nibali L, Suvan J, Lessem J (2006). Periodontal infections cause changes in traditional and novel cardiovascular risk factors: results from a randomized controlled clinical trial.. Am Heart J.

[14] Ramirez-Tortosa MC, Quiles JL, Battino M, Granados S, Morillo JM (2010). Periodontitis is associated with altered plasma fatty acids and cardiovascular risk markers.. Nutr Metab Cardiovasc Dis.

[15] Rufail ML, Schenkein HA, Koertge TE, Best AM, Barbour SE (2007). Atherogenic lipoprotein parameters in patients with aggressive periodontitis.. J Periodont Res.

[16] Seymour GJ, Gemmell E, Reinhardt RA, Eastcott J, Taubman MA (1993). Immunopathogenesis of chronic inflammatory periodontal disease: cellular and molecular mechanisms.. J Periodont Res.

[17] Blessing E, Campbell LA, Rosenfeld ME, Chough N, Kuo CC (2001). *Chlamydia pneumoniae* infection accelerates hyperlipidemia induced atherosclerotic lesion development in C57BL/6J mice.. Atherosclerosis.

[18] Maekawa T, Takahashi N, Honda T, Yonezawa D, Miyashita H (2009). *Porphyromonas gingivalis* antigens and interleukin-6 stimulate the production of monocyte chemoattractant protein-1 via the upregulation of early growth response-1 transcription in human coronary artery endothelial cells.. J Vasc Res.

[19] Liao H, Langmann T, Schmitz G, Zhu Y (2002). Native LDL upregulation of ATP-binding cassette transporter-1 in human vascular endothelial cells.. Arterioscler Thromb Vasc Biol.

[20] Norata GD, Ongari M, Uboldi P, Pellegatta F, Catapano AL (2005). Liver X receptor and retinoic X receptor agonists modulate the expression of genes involved in lipid metabolism in human endothelial cells.. Int J Mol Med.

[21] Tang C, Oram JF (2009). The cell cholesterol exporter ABCA1 as a protector from cardiovascular disease and diabetes.. Biochim Biophys Acta.

[22] Castrillo A, Joseph SB, Vaidya SA, Haberland M, Fogelman AM (2003). Crosstalk between LXR and toll-like receptor signaling mediates bacterial and viral antagonism of cholesterol metabolism.. Mol Cell.

[23] Domon H, Honda T, Oda T, Yoshie H, Yamazaki K (2008). Early and preferential induction of IL-1 receptor-associated kinase-M in THP-1 cells by LPS derived from *Porphyromonas gingivalis*.. J Leukoc Biol.

[24] Zelcer N, Hong C, Boyadjian R, Tontonoz P (2009). LXR regulates cholesterol uptake through Idol-dependent ubiquitination of the LDL receptor.. Science.

[25] Li L, Thompson PA, Kitchens RL (2008). Infection induces a positive acute phase apolipoprotein E response from a negative acute phase gene: role of hepatic LDL receptors.. J Lipid Res.

[26] Lambert G, Charlton F, Rye KA, Piper DE (2009). Molecular basis of PCSK9 function.. Atherosclerosis.

[27] Feingold KR, Moser AH, Shigenaga JK, Patzek SM, Grunfeld C (2008). Inflammation stimulates the expression of PCSK9.. Biochemical and biophysical research communications.

[28] Frikke-Schmidt R, Nordestgaard BG, Stene MC, Sethi AA, Remaley AT (2008). Association of loss-of-function mutations in the ABCA1 gene with high-density lipoprotein cholesterol levels and risk of ischemic heart disease.. JAMA.

[29] Gonzalez-Baro MR, Lewin TM, Coleman RA (2007). Regulation of triglyceride metabolism. II. Function of mitochondrial GPAT1 in the regulation of triacylglycerol biosynthesis and insulin action.. Am J Physiol Gastrointest Liver Physiol.

[30] Schultz JR, Tu H, Luk A, Repa JJ, Medina JC (2000). Role of LXRs in control of lipogenesis.. Genes Dev.

[31] Chen G, Liang G, Ou J, Goldstein JL, Brown MS (2004). Central role for liver X receptor in insulin-mediated activation of Srebp-1c transcription and stimulation of fatty acid synthesis in liver.. Proc Natl Acad Sci U S A.

[32] Shimazaki Y, Saito T, Yonemoto K, Kiyohara Y, Iida M (2007). Relationship of metabolic syndrome to periodontal disease in Japanese women: the Hisayama Study.. J Dent Res.

[33] Yamazaki K, Honda T, Domon H, Okui T, Kajita K (2007). Relationship of periodontal infection to serum antibody levels to periodontopathic bacteria and inflammatory markers in periodontitis patients with coronary heart disease.. Clin Exp Immunol.

[34] Khovidhunkit W, Kim MS, Memon RA, Shigenaga JK, Moser AH (2004). Effects of infection and inflammation on lipid and lipoprotein metabolism: mechanisms and consequences to the host.. J Lipid Res.

[35] Matsushima Y, Hayashi S, Tachibana M (1999). Spontaneously hyperlipidemic (SHL) mice: Japanese wild mice with apolipoprotein E deficiency.. Mamm Genome.

[36] Matsushima Y, Sakurai T, Ohoka A, Ohnuki T, Tada N (2001). Four strains of spontaneously hyperlipidemic (SHL) mice: phenotypic distinctions determined by genetic backgrounds.. J Atheroscler Thromb.

[37] Baker PJ, Evans RT, Roopenian DC (1994). Oral infection with *Porphyromonas gingivalis* and induced alveolar bone loss in immunocompetent and severe combined immunodeficient mice.. Arch Oral Biol.

[38] Ashimoto A, Chen C, Bakker I, Slots J (1996). Polymerase chain reaction detection of 8 putative periodontal pathogens in subgingival plaque of gingivitis and advanced periodontitis lesions.. Oral Microbiol Immunol.

[39] Paigen B, Morrow A, Holmes PA, Mitchell D, Williams RA (1987). Quantitative assessment of atherosclerotic lesions in mice.. Atherosclerosis.

[40] Venkateswaran A, Laffitte BA, Joseph SB, Mak PA, Wilpitz DC (2000). Control of cellular cholesterol efflux by the nuclear oxysterol receptor LXRα.. Proc Natl Acad Sci U S A.

